# Dynamic regulation and functions of mRNA m6A modification

**DOI:** 10.1186/s12935-022-02452-x

**Published:** 2022-01-29

**Authors:** Shanshan Wang, Wei Lv, Tao Li, Shubing Zhang, Huihui Wang, Xuemei Li, Lianzi Wang, Dongyue Ma, Yan Zang, Jilong Shen, Yuanhong Xu, Wei Wei

**Affiliations:** 1 Department of Clinical Laboratory, The First Affiliated Hospital of Anhui Medical University, 218 Jixi Road, Hefei, 230032 Anhui China; 2 Institute of Clinical Pharmacology, Anhui Medical University, Key Laboratory of Anti-Inflammatory and Immune Medicine, Ministry of Education, Anhui Collaborative Innovation Center of Anti-Inflammatory and Immune Medicine, Anhui Anti-Inflammatory and Immune Medicine Innovation Team, Hefei, 230032 Anhui China; 3 The Key Laboratory of Microbiology and Parasitology of Anhui Province, The Key Laboratory of Zoonoses of High Institutions in Anhui, Anhui Medical University, Hefei, 230032 Anhui China

**Keywords:** m6A, mRNA metabolism, Mechanism, Gene expression

## Abstract

*N*^6^-Methyladenosine (m6A), the most abundant internal modification associated with eukaryotic mRNAs, has emerged as a dynamic regulatory mechanism controlling the expression of genes involved in many physiological activities by affecting various steps of mRNA metabolism, including splicing, export, translation, and stability. Here, we review the general role of m6A, highlighting recent advances related to the three major types enzymes that determine the level of m6A modification (i.e., writers, erasers, and readers) and the regulatory mechanism by which m6A influences multiple stages of RNA metabolism. This review clarifies the close connection and interaction between m6A modification and nuclear gene expression, and provides key background information for further studies of its roles in numerous physiological and pathophysiological processes. Among them, perhaps the most eye-catching process is tumorigenesis. Clarifying the molecular mechanism of tumorigenesis, development and metastasis in various tissues of the human body is conducive to curbing out-of-control cell activities from the root and providing a new strategy for human beings to defeat tumors.

## Background

**Fig. 1:**
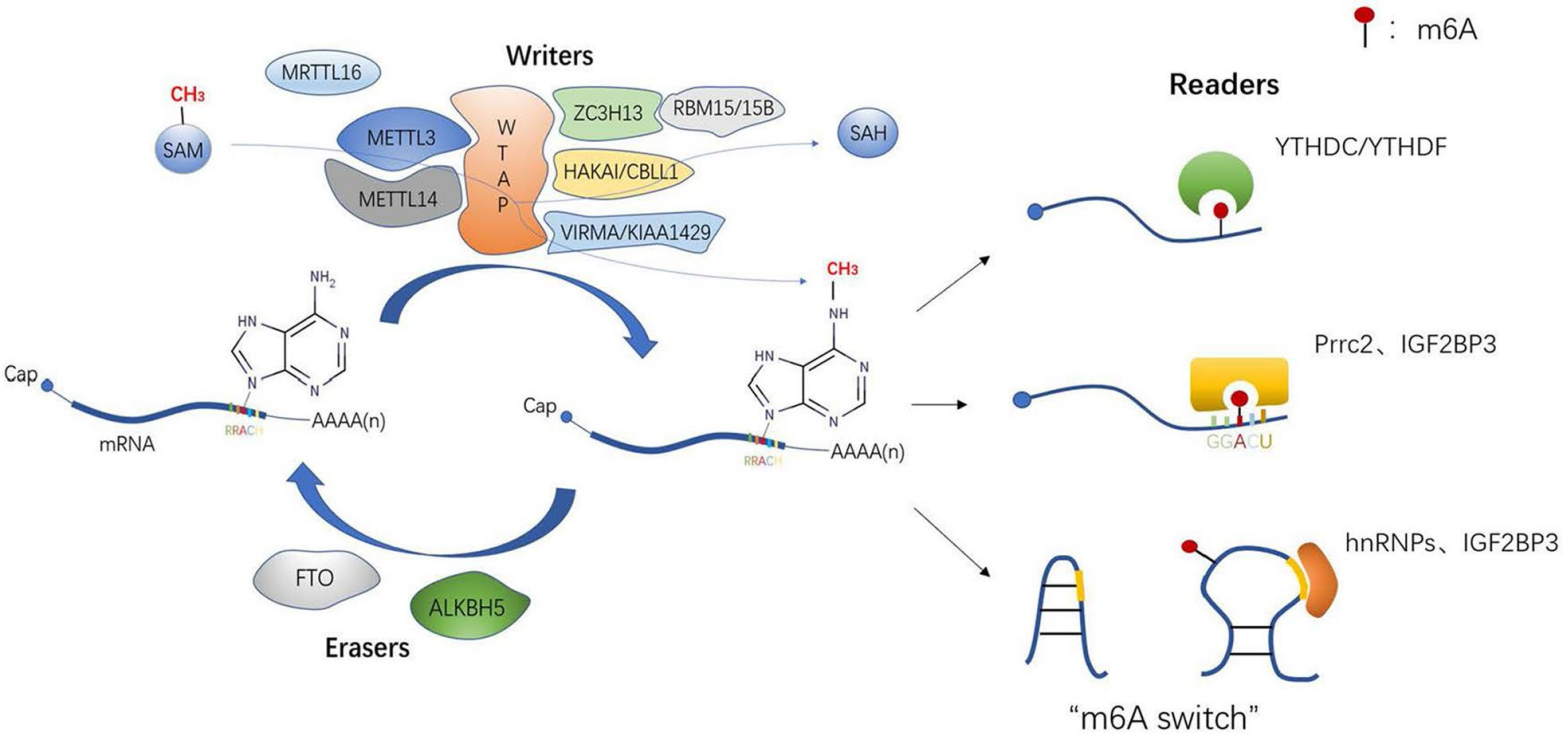
Dynamic and reversible process of m6A modification and three recognition methods of readers

There are more than 170 chemical modifications in RNA. Among these, *N*^6^-Methyladenosine (m6A) is methylation at the *N*^6^ position of adenosine and has been considered as the most abundant and conserved internal transcriptional modification within eukaryotic RNAs. This review focuses on the effects of m6A modification on mRNA metabolism. In early works, RNA m6A was found on the consensus RNA motif RRACH (R = A or G; H = A, U, or C) with enrichment near stop codons and 3′ untranslated regions (UTRs). At the molecular level, it is added by a multicomponent methyltransferase complex, removed by two m6A demethylases, and recognized by an expanding set of m6A binding proteins, and these are termed writers, erasers, and readers, respectively (Table 1). These enzymes enable the process of m6A modification to be dynamic and reversible (Fig. 1). It is possible to specifically interfere with m6A formation by knocking out or overexpressing these enzymes. Thus, it is essential to explore new enzymes involved in this process. In this review, we describe known writers, erases, and readers and summarize recent progress in our understanding of their effects on mRNA metabolism. This information is expected to provide high value to researchers deciphering the links between m6A and human diseases such as cancer.

**Table 1:** m6A regulators in mRNAs

Writer	Eraser	Reader
METTL3	FTO	YTHDC1
METTL14	ALKBH5	YTHDC2
WTAP		YTHDF1/YTHDF2/YTHDF3
VIRMA/KIAA1429		IGF2BP1/IGF2BP2/IGF2BP3
RBM15/15B		hnRNP C/hnRNP G/hnRNP A2/B1
ZC3H13		METTL3
CBLL1/HAKAI		eIF3h
METTL16		Prrc2a
		FMRP
		Ribosome

## Effectors in the m6A pathway

### Writers

In mammals, m6A is deposited in mRNAs by a multi-subunit writer complex with the stable heterodimer core complex METTL3–METTL14 [1]. Methyltransferase-like 3 (METTL3) is an *S*-adenosyl methionine (SAM)-binding protein with methyltransferase capacity, and methyltransferase-like 14 (METTL14) is an allosteric activator that binds to target RNAs [2–4]. Based on the crystal structure of the METTL3–METTL14 complex, METTL14 is catalytically inactive, as evidenced by its lack of a SAM-binding site [2–4]. As critical RNA methyltransferases, METTL3 inhibitors include the universal nucleoside analogue sinefungin and the natural cofactor product *S*-adenosyl-homocysteine (SAH). Recently, more potent and selective bisubstrate inhibitors BA2 and BA4 have been designed that simultaneously exploit the nucleic acid substrate and SAM cofactor binding pockets [5, 6]. In addition, further studies have discovered a handful of additional components associated with the complex and elucidated the mechanisms by which they contribute to the formation of m6A modification. Wilms tumor 1-associated protein (WTAP) interacts with METTL3–METTL14 and is required for localization to nuclear speckles and the recruitment of target RNAs [7]. Vir-like m6A methyltransferase-associated (VIRMA, originally known as KIAA1429) interacts with WTAP and mediates preferential mRNA methylation in 3′ UTRs and near stop codons [8, 9]. RNA binding motif protein 15/15B (RBM15/15B) binds to U enrichment regions and may facilitate the methylation of certain RNAs by interacting with METTL3 in a WTAP-dependent manner [10]. Zinc-finger CCCH-type-containing 13 (ZC3H13) binds to RBM15/15B and links it to WTAP to promote the nuclear localization of the writer complex [11, 12]. Oncogene-like protein 1 (CBLL1), also known as the E3 ubiquitin ligase HAKAI, is required for m6A methylation and functions via interaction with WTPA in *Arabidopsis*; however, its role in mammals is still unclear [8, 13]. Recently, another U6 snRNA methyltransferase, METTL16, was reported to catalyze the addition of m6A in U6-like sequences in *MAT2A*, which encodes SAM synthetase expressed in most cells [14]. Human METTL16 consists of an N-terminal methyltransferase domain (MTD) and a C-terminal vertebrate conserved region (VCR), which cooperatively facilitate m6A43 U6 snRNA modification [15]. Because snRNAs mediate pre-mRNA splicing, METTL16 could likewise affect splicing.

Overall, the METTL3–METTL14 complex and METTL16 are the two most important methyltransferases in m6A modification. Most newly discovered writers are devoted to assisting the METTL3-METTL14 complex to realize methylation more accurately and rapidly. Meanwhile, given that METTL16 only plays the role of writer in maintaining SAM homeostasis, more methyltransferases may be found in specific biochemical activities in the future.

### Erasers

The m6A erasers, including the demethylases fat mass and obesity-associated protein (FTO) and alkb homologue 5 (ALKBH5), oxidatively demethylate m6A modification on RNA [16, 17].

As the first identified RNA demethylase, FTO was initially reported to catalyze the demethylation of m6A in mRNA [16]. However, a large number of subsequent studies have not identified the sequence-specific functional site of FTO. Accordingly, subsequent research has focused on other types of modifications, ultimately demonstrating that FTO has substantially higher catalytic activity for demethylating N^6^,2-*O*-dimethyladenosine (m6A_m_) than for m6A [18]. m6A_m_ has an identical chemical structure in the base moiety to m6A and is found on the second base adjacent to the m7G 5′ cap (cap-m6A_m_) in a portion of mRNAs [19]. Wei et al. have shown that FTO mediates internal m6A and cap-m6A_m_ demethylation of polyadenylated RNA with differential substrate preferences in the nucleus versus the cytoplasm, in which internal m6A demethylation is correlated with transcript-level changes [20]. FTO regulates gene expression by altering patterns of alternative splicing and translation. The highly expressed FTO and its prevalent m6A demethylation play oncogenic roles in various cancers, especially acute myeloid leukemia. Strong evidence suggests that FTO promotes leukemic oncogene-mediated cell transformation and leukemogenesis and inhibits the ATRA-mediated differentiation of leukemic cells by decreasing stability in the mRNA transcripts of its critical target genes, such as ASB2 and RARA, thereby triggering corresponding signaling cascades [21]. ASB2 and RARA have been reported to be upregulated during normal hematopoiesis and in all-trans-retinoic acid (ATRA)-induced differentiation of leukemia cells. They are also reported to function as key regulators during these processes [22–26]. Thus, several studies have evaluated the application of FTO inhibitors, with the aim of finding highly effective therapies. In recent years, a series of specific or non-specific FTO inhibitors, such as rhein, meclofenamic acid (MA), MO-I-500, fluorescein, and R-2-hydroxyglutarate (R-2HG), have been identified [27–31]. Nevertheless, the clinical value of these inhibitors is limited by their weak biological function and low sensitivity and/or specificity. This led to the discovery of two other MA-derived FTO inhibitors, FB23 and FB23-2 [32]; however, these also show an unsatisfactory degree of inhibition. CS1 and CS2 specifically target FTO and efficiently suppress m6A demethylase activity by occupying the catalytic pocket and interfering with the binding of FTO to m6A-modified RNAs [33]. Future research should focus on clarifying the precise mechanisms by which m6A modification contributes to the occurrence and development of various refractory tumors and finding efficient therapeutic targets.

For the second identified demethylase ALKBH5, m6A is the only known substrate to date [17]. Unlike FTO, ALKBH5 has no activity towards m6A_m_ and is a nuclear protein with localization in nuclear speckles. In vitro [16, 27], ALKBH5 is enriched in the testes and an ALKBH5 deficiency in male mice results in spermatogenesis defects that impaired fertility [17]. In addition, an ALKBH5 deficiency leads to defective cerebellar development in mice exposed to hypobaric hypoxia by altering the original m6A level and decreases the production of itaconate, which is required for viral replication, thus conferring resistance to viral challenge [34, 35]. In vivo, specific transcripts and m6A sites in different cells and tissues can mediate the oncogenic effects of aberrant ALKBH5 expression by recruiting diverse downstream molecules. For example, ALKBH5 inhibits tumor growth and metastasis by reducing the expression and activity of YAP in non-small cell lung cancer [36]. As the main component of the Hippo signaling pathway (an inhibitory pathway that hinders cell growth and controls cell proliferation, organ size, and homeostasis), dephosphorylated YAP promotes target genes transcription, which controls organ size, tumor cell proliferation, and metastasis [37, 38]. It also suppresses hepatocellular carcinoma (HCC) by inhibiting LY6/PLAUR Domain Containing 1 (LYPD-1) expression, which has been identified as a novel oncoprotein in HCC [39]. Finally, ALKBH5-mediated m6A demethylation prevents the growth and metastasis of pancreatic cancer by the post-transcriptional activation of PER1 in an m6A-YTHDF2-dependent manner [40]. These findings imply that ALKBH5 acts as a tumor suppressor. Yet, overexpressed ALHBK5 up-regulates critical target TACC3, a prognosis-associated oncogene in various cancers, to selectively promote tumorigenesis and cancer stem cell self-renewal in acute myeloid leukemia [41].

In summary, the existence of demethylase FTO and ALKBH5 makes m6A modification a reversible process, but their different substrates and organ enrichment preferences lead them to participate in different biological pathways. Furthermore, when the expression level of FTO or ALKBH5 is altered or dysfunctional, it may be involved in the occurrence and progression of a variety of tumors as a tumor suppressor gene or oncogene in an m6A-dependent manner.

### Readers

m6A readers determine the fate of target mRNAs by identifying and interpreting m6A sites on diverse transcripts. Reader proteins are divided into direct and indirect readers according to their ability to directly and specifically combine with m6A, although the recognition mechanism has not been determined for some readers.

Direct readers include five YTH-containing proteins categorized into three classes: YTHDC1/DC1, YTHDC2/DC2, and YTHDF (YTHDF1/DF1, YT-HDF2/DF2, and YTHDF3/DF3). Previous studies have suggested that the YTH domain binds to transcripts in an m6A-dependent manner [42]. Moreover, we found that catalytically inactive METTL3 interacts with eukaryotic translation initiation factor 3 subunit h (eIF3h) to enhance translation when tethered to reporter mRNA at m6A sites close to the stop codon [43]. eIf3 directly binds to the m6A site in the 5ʹ UTR to drive translation [44]. In addition to acting as a methyltransferase to deposit m6A, METTL3 also functions as a direct reader together with eIF3 to identify m6A-containing transcripts. In addition, a recent study of HEK293 cells provided in vitro evidence that coding sequence (CDS) m6A methylation leads to ribosome pausing at the A site in mammalian cells [45]. These findings suggested that ribosomes act as direct readers to recognize m6A methylation; however, the exact mechanism is still unclear. Recently, a new type of RNA-binding protein (RBP) has emerged. in particular, in the process of oligodendroglial specification and myelination, a novel m6A specific binding protein family, including Prrc2a and Prrc2c in neural cells, specifically binds to a consensus GGm6ACU motif via a new Prrc2a domain (named GRE domain) to stabilize the critical transcript [46]. It is not clear whether Prrc2 in other organs contributes to the regulation of biological activities.

With respect to indirect readers, heterogeneous nuclear ribonucleoproteins (hnRNPs) include hnRNPC, hnRNPG, and hnRNPA2/B1. Each hnRNP contains at least one RNA-binding domain, such as the RNA recognition motif, K-homology domain, or arginine/glycine-rich box [47]. These proteins cannot specifically identify m6A-modified sites, except by a “m6A switch” mechanism in which m6A induces RNA unfolding and increases the accessibility of hnRNPs to single-stranded RNA [48]. Instead of directly binding to m6A, the m6A switch may promote the affinity of hnRNP A2/B1 to certain adjacent binding sites [49]. Similarly, a recent data analysis has indicated that hnRNPC acts as an m6A reader not by recognizing the N6-methyl group but rather by binding to a purine-rich motif that becomes unpaired and accessible upon nearby m6A modification [50]. This indicates that the binding efficiency for the surrounding sequences and RBPs is affected by m6A by changing the structure of RNA. However, it is not clear whether other m6A-binding proteins, such as FMRP, the fragile X mental retardation protein, and insulin-like growth factor 2 mRNA-binding proteins (IGF2BPs; including IGF2BP1, IGF2BP2, and IGF2BP3) bind to m6A directly. Current studies only show that FMRP interacts with YTHDF2 and thereby identifies m6A [51]. IGF2BP3 can not only directly recognize m6A via a GGAC motif, but can also act in a manner dependent on the m6A-structural switch [50, 52]. Therefore, further studies of specific identification methods are needed.

Another interesting observation from pulldown experiments is that many proteins are repelled by the m6A modification, indicating that the binding of these proteins to RNA is inhibited when the adenosine base is methylated. The two most consistently and strongly repelled proteins are the stress granule proteins G3BP1 and G3BP2 (G3BPs), which are repelled in an RNA sequence context-dependent manner and stabilize targets via ribonucleoprotein granules (RNPs) [53]. Likewise, once the m6A residue is lost, it recruits ELAV-like RNA binding protein 1 (ELAVL1, also known as HuR) to distant HuR-binding sites to facilitate protein expression. For instance, in glioblastoma stem-like cells, only ALKBH5-mediated unmethylated nascent transcripts of *FOXM1* can interact with HuR to enhance FOXM1 expression and then promote self-renewal and tumorigenesis [54]. The “RNA-binding regulatory peptide” (RBRP) encoded by the lncRNA LINC00266-1 is an additional regulatory subunit of m6A readers and strengthens m6A recognition by interacting with the m6A reader IGF2BP1 to exert oncogenic functions [55]. The dysregulation of lncRNAs indeed contributes to various cancers and may be a working mechanism. The wide distribution of regulatory subunits of m6a readers in cells and variety of biological functions reveals an additional regulatory layer of the m6A pathway and m6A recognition: in addition to various protein, other types of RNA can also participate in the identification of m6A modification on mRNA.

In conclusion, readers recognize m6A modifications in three main ways (Fig. 1): 1. they directly combine with m6A sites; 2. they specifically bind to a consensus GGm6ACU motif; and 3. they fully expose the internal m6A site by opening the “m6A switch”. Whether directly or indirectly, a variety of recognition methods maximize the potential biological role of m6A.

## m6A methylation regulates mRNA metabolism

mRNA metabolism involves alternative splicing, nuclear export, translation, and degradation. As the most common and extensive base modification method at the RNA level, m6A methylation profoundly influences all aspects of mRNA-associated processes. Considering that all steps of RNA metabolism are inherited and closely connected, for m6A, interference with even single step has important downstream consequences. Post-transcriptional modification precisely controls the expression of diverse genes, thereby affecting individual growth, development, and many other biological functions.

### mRNA alternative splicing

**Fig. 2:**
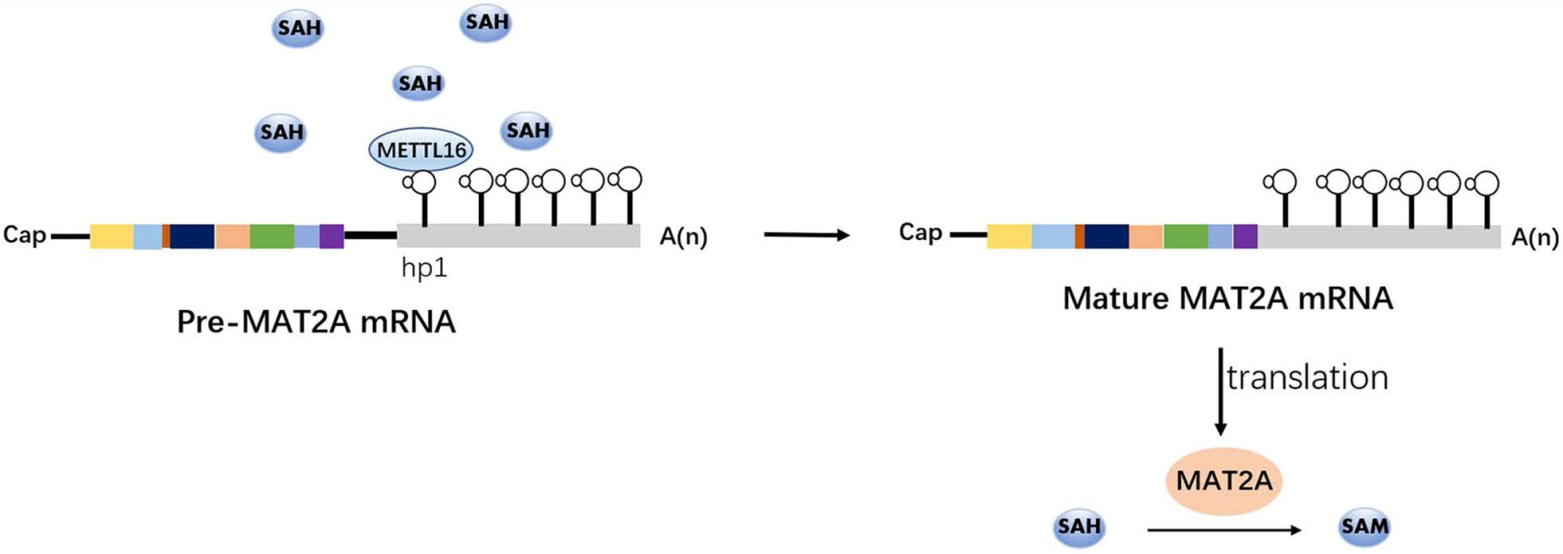
METTL16-mediated regulation of MAT2A synthesis by environmental factors

Nascent transcripts synthesized from DNA must undergo alternative splicing before transformation into mature transcripts with biological functions. m6A modification regulates gene expression by interfering with this process. m6A methylation that directly influences splicing is usually located near exonic or intronic splice junctions, matching its function. Therefore, detecting m6A redundancy at these positions has become one of the main methods for predicting whether the m6A modification regulates pre-mRNA alternative splicing. In METTL3-knockout embryonic cells, the fraction of known alternatively spliced exons showed altered splicing compared with METTL3-knockout ES cells, which suggests that m6A has little effect on pre-mRNA splicing [56, 57]. Despite a low frequency of METTL3-dependent alternative splicing events, the newly described m6A-regulated splicing is rapid and dynamic in changing environments and under pathological conditions. It only occurs under specific circumstances, rather than functioning as a wide-ranging regulatory event that persists under normal physiological conditions [14, 58, 59]. Thus, the frequency of splicing events varies depending on environmental changes, although these fluctuations may be small. For example, innate immunity and endoplasmic reticulum (ER) stress signaling following *Flaviviridae* infections contribute to the loss of m6A in *CIRBP* mRNA, leading to the robust production of CIRBP protein via alternative splicing, which then facilitates viral replication [58]. In lung cancer, RNA binding protein TARBP2-mediated deposition of m6A in target transcripts inhibits efficient splicing to increase the rate of intron retention. It then accelerates unstable transcript-targeted degradation and promotes the growth of the tumor [59]. Furthermore, another methyltransferase, METTL16, rapidly induces the splicing of the intron of *MAT2A*, encoding a SAM synthetase, and maintains low levels of intracellular SAM. Under SAM-limiting conditions, the absence of a methyl donor obstructs methylation reactions on hp1 of MAT2A, which drives efficient splicing by increasing METTL16 occupancy on hp1 to increase MAT2A expression [14] (Fig. 2). These results support those of previous studies showing that the number of m6A sites tends to be higher for transcripts that undergo dynamic regulation than for stable housekeeping mRNAs [60], further proving that m6A-mediated splicing events are likely to occur disproportionately in transcripts able to respond to environmental changes. Hence, to determine the extent to which m6A functions in mRNA splicing, it is necessary to elucidate the precise mechanisms underlying the m6A-dependent regulation of alternative splicing.

**Fig. 3:**
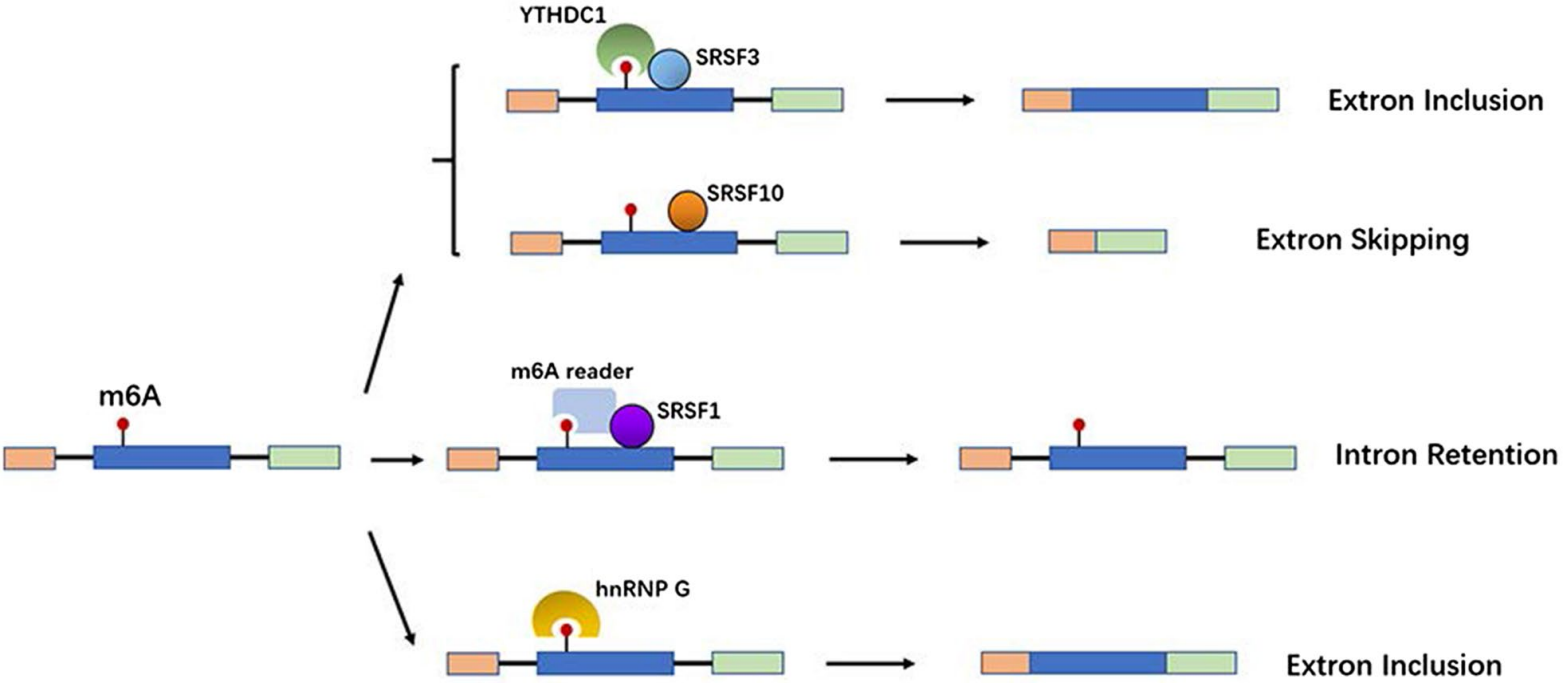
Readers recruit splicing factors to control the alternative splicing of nascent transcripts

Despite multiple studies demonstrating that methyltransferases, demethylases, and readers located in nuclear speckles cause alternative splicing, including exon inclusion and exon skipping, little is known about the specific mechanism underlying these effects [61–64]. A common mechanism underlying the regulation of splicing involves the recruitment of various splicing factors. The m6A reader YTHDC1 recruits the splicing factor SRSF3 to promote exon inclusion but antagonizes *SRSF10* mRNA binding, which facilitates exon skipping [63]. As a promoter of lung cancer growth, TARBP2 recruits the methyltransferase complex to deposit m6A marks on transcripts, thereby resulting in intron retention with the help of the splicing factor SRSF1 [59]. In a special phenomenon, reader proteins or methyltransferases recruited by m6A, such as METTL16-VCRs and hnRNPG, themselves act as effective splicing factors [14, 64] (Fig. 3). Since splicing factors play a critical role in this process, it is reasonable to speculate that m6A may indirectly regulate the alternative splicing of target mRNAs by affecting the expression of splicing regulators.

### mRNA nuclear export

After nuclear processing, mature mRNA enters the cytoplasm from the nucleus for translation. m6A modification is also involved in this process; in essence, this kind of regulation utilizes the formation of steric resistance to ultimately target translation. In general, the hypermethylation of specific sites on the transcript may enhance nuclear export. After viral infection, the RNA helicase DDX46 inhibits antiviral innate responses by entrapping selected antiviral transcripts in the nucleus by recruiting ALKBH5 to demethylate the m6A-modified target transcripts [65]. In contrast, the nuclear export of hypermethylated RNAs is enhanced in the cerebellum of Alkbh5-deficient mice exposed to hypobaric hypoxia [34]. In blood malignancies, the nuclear speckle-specific long noncoding RNA MALAT1 hijacks fusion proteins and chimeric mRNA to promote chimeric mRNA export in an m6A-dependent manner by mediating the colocalization of oncogenic fusion proteins with the METTL3-METTL14 complex, thus suppressing hematopoietic cell differentiation [66]. This provides a mechanism for the spatiotemporal regulation of gene expression during development.

**Fig. 4:**
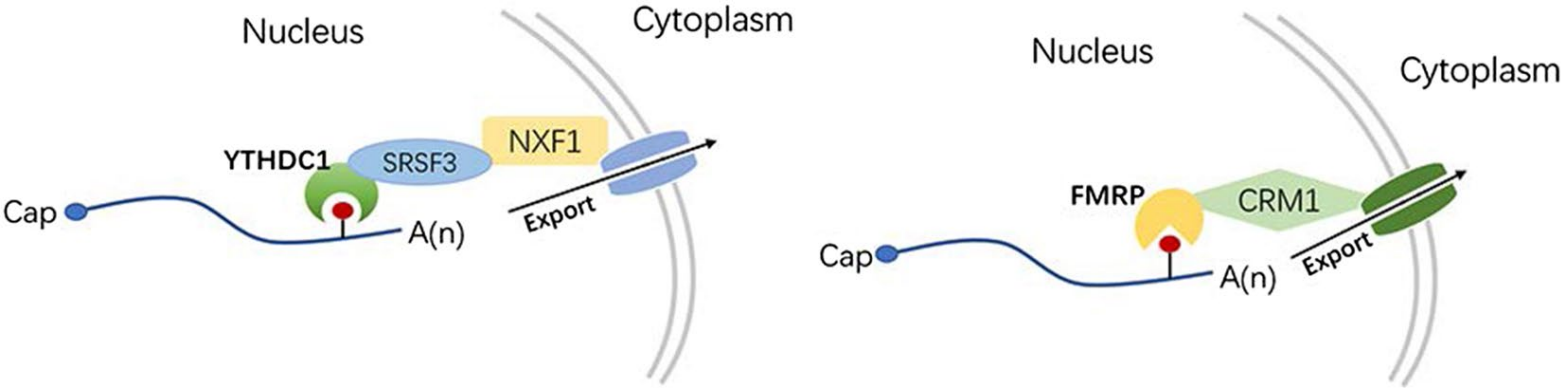
Readers mediate mRNA export through channel proteins

m6A methylation facilitates the nuclear export of mature transcripts by binding to *YTHDC1* or *FMRP*. The methylated mRNA is recognized by the nuclear protein YTHDC1 and delivered to the nuclear mRNA export receptor NXF1 via interactions with the splicing factor and nuclear export adaptor protein SRSF3 [67]. Another newly discovered reader protein, FMRP, preferentially binds to m6A-tagged mRNAs to drive nuclear export via the CRM1-dependent export pathway during neural differentiation [68] (Fig. 4). Collectively, the nuclear reader protein-dependent induction of m6A-modified RNAs to approach and pass through the exit channel or pore is crucial.

### mRNA translation

**Fig. 5:**
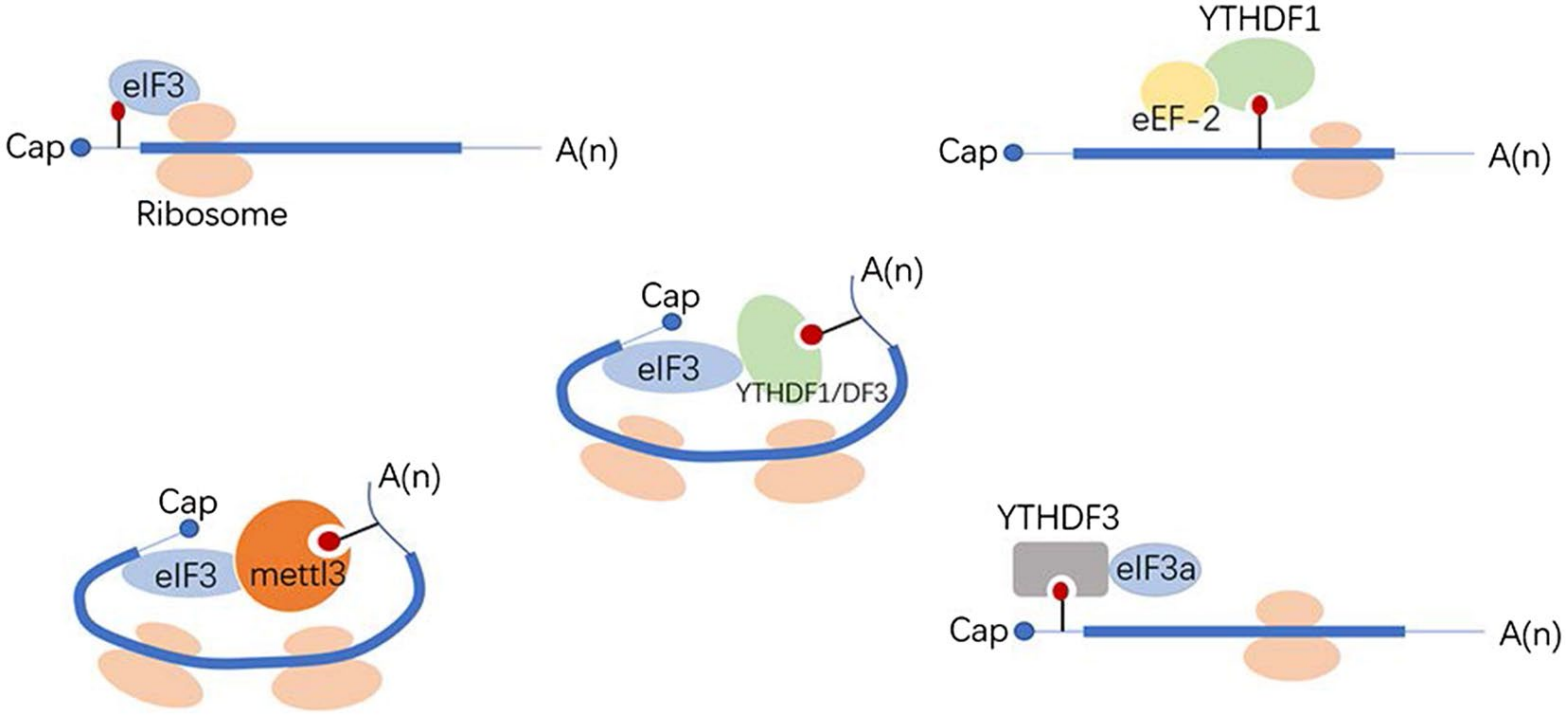
Cap-independent translations

At the molecular level, the mechanism by which m6A modification improves the translation efficiency is mainly dependent on the binding of reader proteins to protein factors required in the translation process, and m6A modifications located in different RNA regions exert effects by various modes of action. METTL3 promotes translation by identifying 5′ UTR m6A and 3′ UTR m6A. In eukaryotic cells, protein synthesis typically begins with the binding of eIF4F to the 7-methylguanylate (m7G) cap found at the 5′ end of the majority of mRNAs. The eIF4F complex consists of (i) eIF4E, a 5′ cap-binding protein; (ii) eIF4A, an RNA helicase; and (iii) eIF4G, a scaffold protein that binds to eIF4E and eIF4A. In lower and higher eukaryotes, DDx6 RNA helicase (DOZI in plasmodium) plays an important regulatory role and inhibits translation by interacting with eIF4E or the activation of the eIF4E repressor 4E-T [69, 70]. However, the 5′ UTR m6A-mediated translation initiation of non-TOP (5′ terminal oligopyrimidine element) mRNAs does not require eIF4F, which instead shows a cap-independent mechanism (Fig. 5) by binding to other initiation factors, such as eIF3, to coordinate with the classical mode together to produce adaptive translatomes in response to environmental and physiological stimuli [71]. In another closed-loop model, METTL3 binds to eIF3, which interacts with mRNA cap-associated proteins, resulting in the formation of an mRNA loop. However, direct METTL3 tethering can promote translation only when bound to the 3′ UTR at a position near the stop codon. This model was proposed to explain the promotion of translation by ribosome recycling [43]. Furthermore, during the epithelial-mesenchymal transition, YTHDF1 mediates the CDS m6A-enhanced translation elongation of Snail mRNA via interactions with the translation elongation factor eEF2, although a previous study indicated that it also binds to *eIF3* in the 3′ UTR [72]. Similarly, YTHDF3 significantly promotes the binding of eIF3a to m6A residues within the 5′ UTR of *YTHDF3* mRNA to enhance cap-independent translation in breast cancer brain metastases [73]. Other readers (e.g., IGF2BP and YTHDF2) have also been reported to enhance translation; however, the precise mechanism has not been clarified [45, 52].

At the cellular level, the deposition of m6A on mRNAs is a co-transcriptional process and depends on the dynamics of RNA polymerase II (RNAPII) transcription, in which the slow progression or frequent pausing of RNAPII may increase the probability of m6A-transferase complex (MTC) engagement. Furthermore, excessive m6A reduces translation efficiency [74]. However, the TATA-box promoter element and CAATT-box promoter element-bound METTL3 induce m6A modification within target mRNAs and present a high translation efficiency by decreasing ribosome stalling [74, 75]. Despite contradictory findings, these studies all demonstrate a general and widespread link between transcription and translation governed by the epigenetic modification of mRNAs.

Genetic analyses have shown that m6A affects translation efficiency in a context-dependent manner and that the downstream effects of m6A are heterogeneous [76]. This is consistent with our previous observations that the loss-of-function of the non-nuclear pool of FTO results in increased m6A modification and decreased local translation of axonal *GAP-43* mRNA, eventually repressing axon elongation [77]. Suboptimal transcription rates lead to elevated m6A contents, thereby resulting in reduced translation [74]. However, studies have mainly reported stimulatory effects of m6A modification on translation. Given that methylation within UTRs typically promotes translation and methylation within CDRs reduces translation, we speculate that the effect of m6A on translation may depend on the location of the modified nucleotide within the transcript. Interestingly, recent data have shown that the depletion of the classical m6A reader YTHDF1 leads to an overall reduction in translation efficiency for transcripts harboring YTHDF1-bound m6A peaks, with the opposite effects for approximately 33% of YTHDF1 targets [76]. This shows that there are still unresolved mechanisms that mediate different effects of m6A. Specific RBPs, such as YBX3, probably mediate the effects of m6A by repressing the translation of YBX3-bound m6A transcripts [76]. Accordingly, additional recognition proteins or m6A-associated RNA regulators with different functions have yet to be discovered. More research is needed to clarify the different effects of m6A on the translation process and the precise underlying mechanisms.

### mRNA stability

**Fig. 6:**
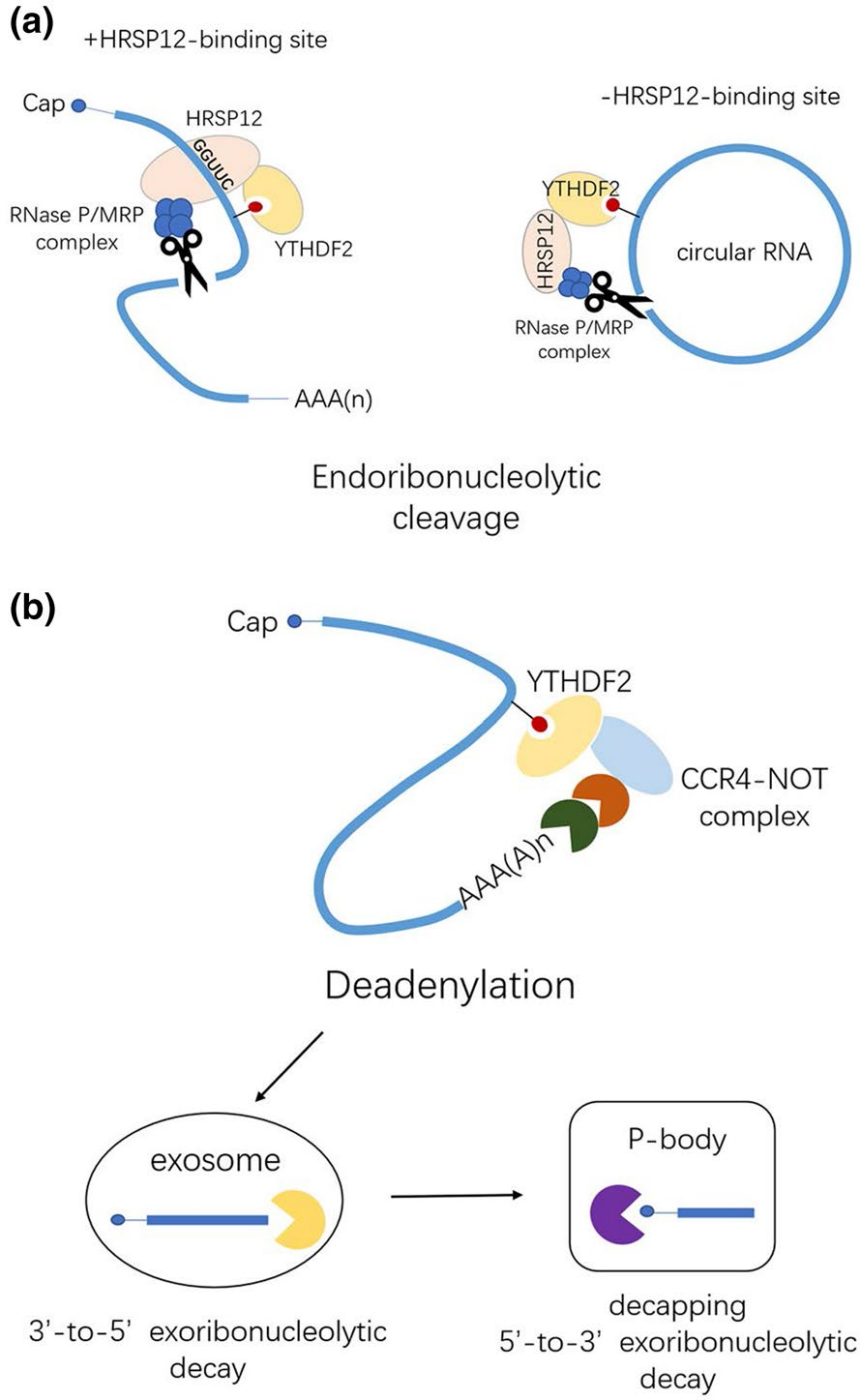
Two main degradation mechanisms of m6A-remarked mRNAs

**Fig. 7:**
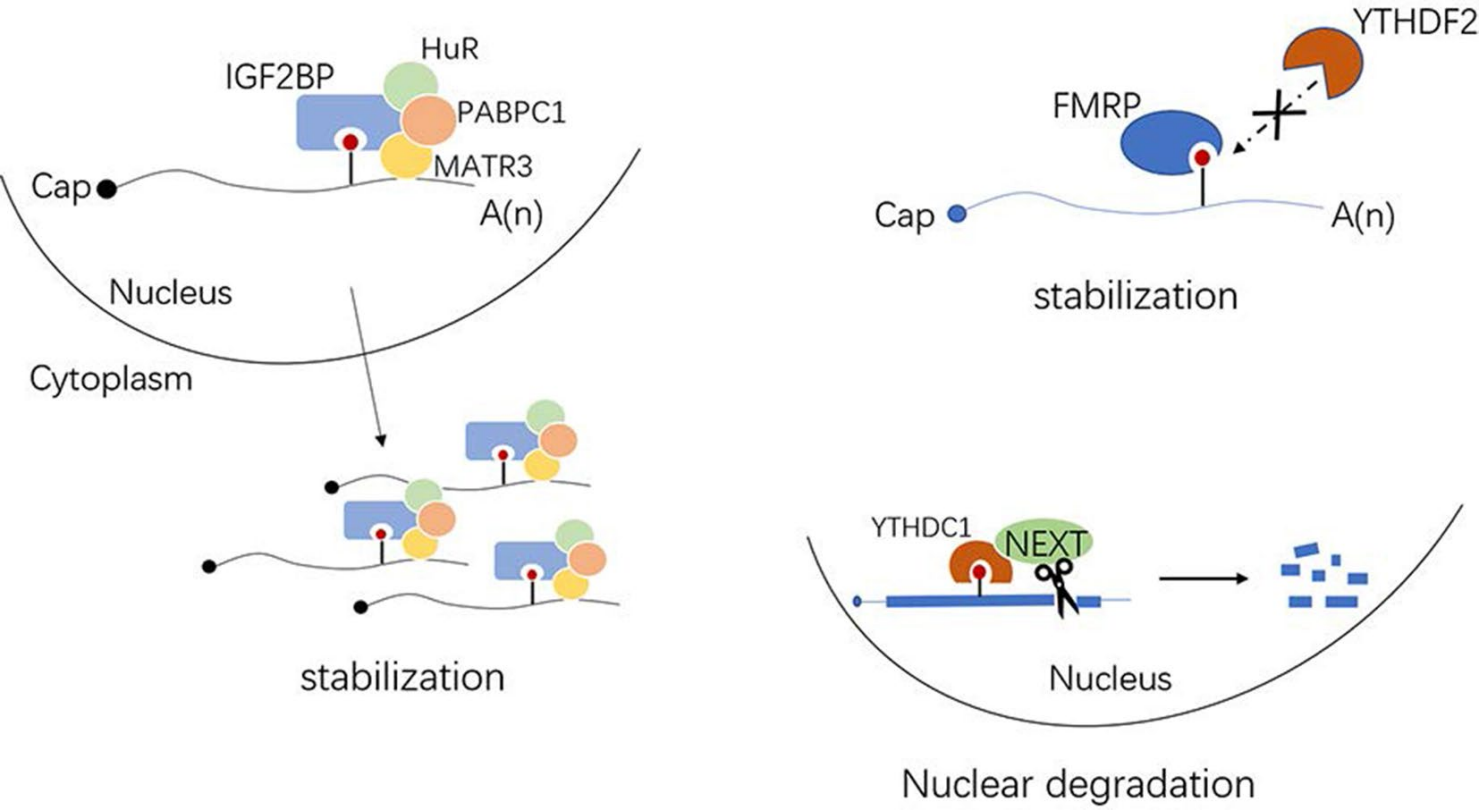
Readers affect the stability of m6A-remarked mRNAs in different ways

m6A-containing transcripts mediate RNA decay via different molecular mechanisms involving the recruitment of reader proteins. Recent reports have consistently indicated that YTHDF2 is the major decay-inducing reader protein. YTHDF2-bound m6A mRNAs are degraded by at least two pathways. First, when a heat-responsive protein (HRSP)12-binding site and an RNase P/MRP (endoribonucleases)-directed cleavage site exist upstream and downstream of the YTHDF2-binding site, respectively, HRSP12 functions as an adaptor to bridge YTHDF2 and RNase P or MRP, eliciting the rapid degradation of YTHDF2-bound RNAs by an endoribonucleolytic cleavage pathway [78]. Second, YTHDF2 directly recruits the CCR4/NOT deadenylase complex to trigger deadenylation and subsequently initiates the degradation of m6A-containing mRNA via exosomes (3′-to-5′ exoribonuclease complex) and P bodies where the decapping complex and 5′-to-3′ exoribonuclease (XRN1) are enriched [79–82]. The CCR4-NOT complex is a nine-subunit complex containing two deadenylase subunits, CAF1 (or its paralogue POP2) and CCR4A (or its paralogue CCR4B) [83]. A subset of sites lacking circRNAs is also associated with YTHDF2 in an HRSP12-dependent manner and is selectively downregulated by RNase P or MRP. It has been proposed that two decay patterns mediated by the HRSP12-RNase P/MRP or CCR4-NOT complex may combine leading to efficient m6A RNA decay [78] (Fig. 6). Moreover, IGF2BPs stabilize mRNAs by binding to RNA stabilizers, such as HuR, matrin 3 (*MATR3*), and poly(A)-binding protein cytoplasmic 1 (*PABPC1*) [52]. FMRP stabilizes targets by blocking m6A/YTHDF2-mediated degradation, while YTHDC1 facilitates the decay of mRNA targets via Nuclear Exosome Targeting (NEXT) complex-mediated nuclear degradation [51, 84] (Fig. 7). Although growing evidence suggests that DF2 has a key role in RNA decay, a recently proposed model proposes that YTHDF proteins function together to mediate the degradation of m6A-mRNAs [85]. Interestingly, a recent report has shown that DF2 has the opposite effect, stabilizing RNA in an m6A-dependent manner [86]. It is difficult to determine whether this is a direct or indirect effect of DF2. Furthermore, the change in local RNA structure due to m6A modification may also affect the stability of m6A-containing mRNAs [87]. However, we still have a limited understanding of the detailed mechanism of action of the newly discovered YTHDF2-HRSP12-RNase P/MRP pathway and downstream regulatory. If these two modes of action do not function cooperatively, it is necessary to determine which is more effective and their respective scope of action.

**Fig. 8:**
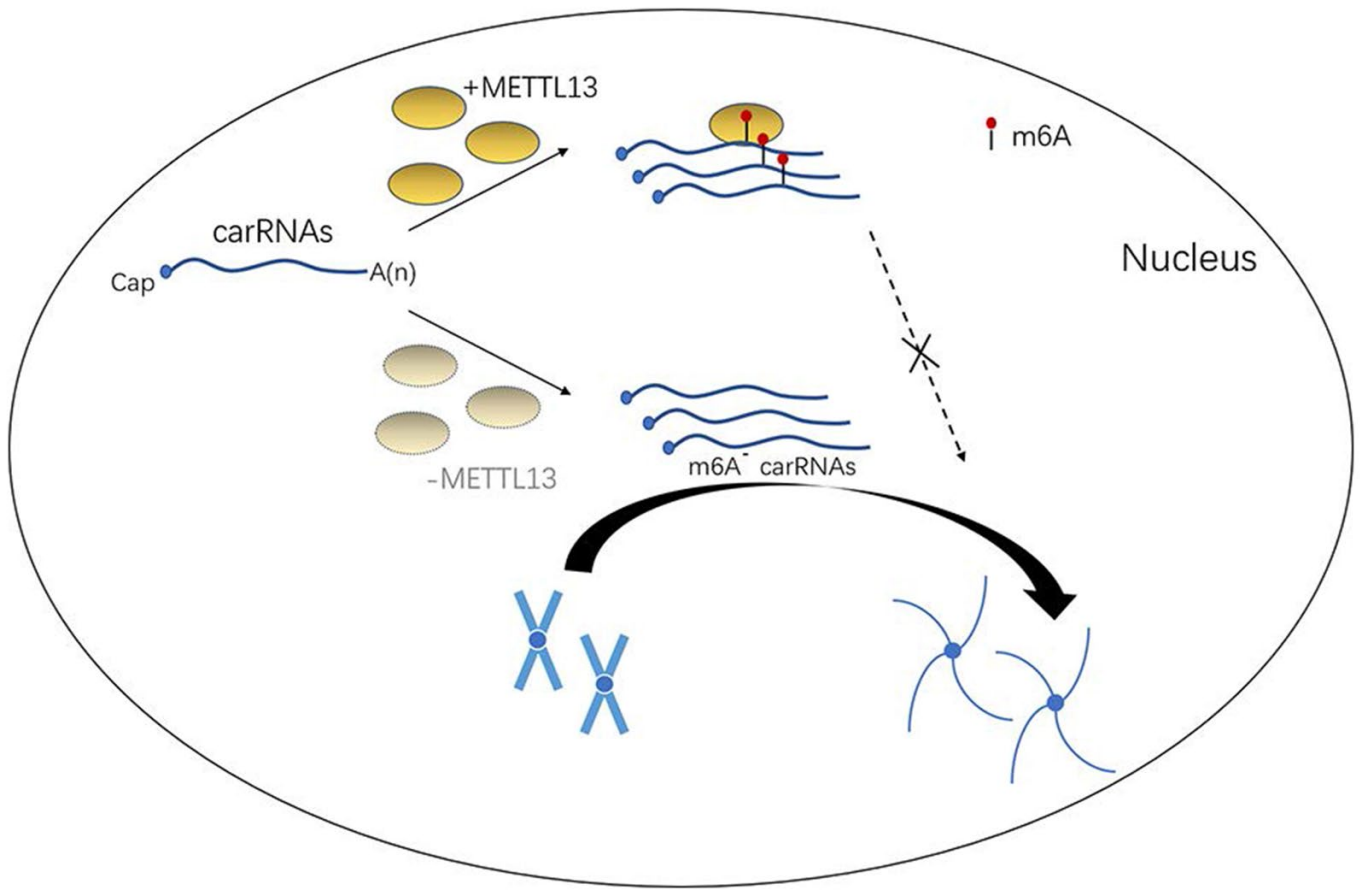
carRNAs globally tune chromatin accessibility and transcription activity

m6A modification-mediated RNA stability is a complicated process that involves crosstalk with other mRNA modifications, RNA species, or decay pathways. Remarkably, transcription dynamics regulate the rate of RNA degradation to buffer mRNA levels. Efficient transcription results in relatively long poly(A) tails, conferring higher mRNA expression stability. In contrast, the impairment of transcription is associated with enhanced m6A deposition, preferential activity of the CCR4-NOT complex, shortened poly(A) tails, and diminished mRNA stability [88]. Overall, to balance mRNA levels, cells effectively inhibit the degradation machinery and promote mRNA stabilization in response to global reductions in transcription [88]. A recent study has shown that the alternative decay of m6A-deposited chromosome-associated regulatory RNAs (carRNAs) globally tunes chromatin accessibility and transcription activity [84] (Fig. 8). This provides strong evidence that the process of RNA degradation is tightly coupled with transcription. Hence, the regulation of m6A at each stage of mRNA metabolism is closely related. Therefore, the effects of m6A on mRNA metabolism should be comprehensively evaluated.

## Conclusions

Many recent studies have reported that the m6A modification regulates individual reproduction, development, diseases, and aging events, emphasizing the extensive influence of m6A methylation at the RNA level. Therefore, a thorough understanding of the detailed mechanisms underlying the effects of m6A methylation has broad applications for our understanding of biological activities and for external interventions. Nevertheless, confusion remains: first, the addition and erasure of m6A methylation are environment-dependent although the biological signals connecting these two processes within the cell are still unclear; second, as reader proteins are the major determinant of the specific effects of m6A modification, it is crucial to characterize events occurring after m6A identification. This is an area that requires substantial attention. In particular, the discovery of m6A-related RBPs that do not directly recognize the m6A methylation site has expanded the species and functions of reader proteins, and the effects of m6A are thereby more heterogeneous. Future research should focus on the discovery of additional special RBPs; third, we have recently found that the transcription rate can influence the translation and stabilization of m6A-modified transcripts and that the expression of special proteins mediated by m6A modification promotes the open chromatin state and downstream transcription. These findings demonstrated that the regulation of gene expression by m6A modification is not limited to the post-transcriptional stage but starts as early as before transcription, prompting us to focus on the effects of m6A modification at a new level—transcription.

## Data Availability

Not applicable.
